# Decoding the genetic architecture of seed quality traits in mungbean through quantitative trait loci mapping

**DOI:** 10.3389/fpls.2026.1890274

**Published:** 2026-08-05

**Authors:** Chunxiu Ren, Lipan Qiao, Jiaqi Qiao, Huimei Sun, Hongjie Li, Bin Li, Xiaopeng Hao, Fan Yang, Dongao Huo

**Affiliations:** 1 College of Biological Sciences and Technology, Taiyuan Normal University, Taiyuan, China; 2 Key Laboratory of Plant Molecular Physiology, Institute of Botany, Chinese Academy of Sciences, Beijing, China; 3 Center for Agricultural Genetic Resources Research, Shanxi Agricultural University, Taiyuan, China

**Keywords:** mungbean, seed quality, RIL, QTL, candidate gene

## Abstract

**Introduction:**

Mungbean [*Vigna radiata* (L.) R. Wilczek] has emerged as a highly valued crop owing to its unique functional properties. Improving seed quality represents a major goal in mungbean breeding; however, the systematic identification of quantitative trait loci (QTLs) governing key seed quality traits remains limited.

**Methods:**

In this study, a recombinant inbred line (RIL) population derived from a cross between Zhonglv1 and HB211 was developed, and phenotypic data were collected across three distinct environments. Three complementary QTL mapping methods—composite interval mapping (CIM), inclusive composite interval mapping (ICIM), and multiple QTL mapping (MQM)—were utilized to detect genomic regions associated with protein, starch, oil, and moisture content.

**Results and discussion:**

In total, 162 QTLs were identified, including 44, 30, 43, and 45 QTLs were identified for protein, starch, oil, and moisture content, respectively. Among these, stable QTLs were consistently detected across multiple environments and methods, highlighting their potential utility in breeding applications. The putative candidate genes underlying these robust QTLs were further annotated, providing preliminary insights into the genetic control of seed quality traits. This study establishes a comprehensive genetic framework for future functional studies and the development of improved mungbean varieties with enhanced seed quality.

## 1 Introduction

Mungbean (*Vigna radiata* (L.) R. Wilczek), a member of the legume family, is a particularly valuable crop due to its unique biological and agronomic traits (Yao et al., 2008; Kim et al., 2015). Like other legumes, it plays a crucial role in sustainable agriculture through biological nitrogen fixation, enriching soil fertility and reducing dependency on synthetic fertilizers (Singh et al., 2016). Notably, mungbean has a short growth cycle, making it an ideal candidate for rotation with cereal crops, and exhibits strong drought tolerance, enhancing its adaptability to challenging environments (Murakami et al., 1991). In recent years, the cultivation area of mungbean has expanded globally, driven by the rising consumer demand for plant-based protein sources (Li et al., 2017). Nutritionally, mungbean is a highly valuable crop, offering a superior protein content (20 - 25%) compared to chickpea while maintaining a lower oil content (1 - 2%) than soybean (Tang et al., 2014). Additionally, mungbean seeds are rich in essential vitamins and minerals. Its high nutrient density positions mungbean as a key crop for supporting sustainable diets worldwide (Pratap et al., 2021).

One of the primary objectives in mungbean breeding programs is the improvement of seed quality traits. Quality variation caused by genetic background in mungbean is closely associated with dynamic changes in multiple metabolic pathways (Zhang et al., 2024a). Seed quality in mungbean is a complex trait primarily determined by protein, starch, oil, and moisture content, which collectively influence its nutritional value and processing suitability. As a high-quality plant protein source, mungbean features a well-balanced amino acid profile with particularly high lysine content that effectively complements the lysine deficiency in cereal-based diets (Idris et al., 2025). Mungbean starch exhibits unique functional properties, including high digestibility, a low glycemic index, and excellent gelling capacity (Englyst et al., 1992; Liu et al., 2006). These characteristics are valuable for both traditional food applications and modern food industries, such as noodle and snack production. In contrast to the extensive oil research on soybean, studies on mungbean oil are scarce. Notably, mungbean oil contain 72.8% unsaturated fatty acids, contributing to its potential health benefits (Dahiya et al., 2015). Additionally, mungbean seeds exhibit a high total tocopherol content, surpassing other legumes such as black gram, pigeon pea, and horse gram, highlighting its antioxidant potential (Gopala Krishna et al., 1997). Seed moisture content critically affects physical and processing traits of mungbean, including grain dimensions, hardness, sphericity, porosity, and bulk density. These properties influence storage stability, milling efficiency, and end-product quality.

Similar genetic studies on seed quality traits have been extensively conducted in other legume crops. Soybean, as one of the most important protein- and oil-producing crops, has been intensively investigated for the genetic basis of seed composition traits. Numerous QTLs associated with seed protein and oil contents have been identified through linkage mapping and genome-wide association studies. Among them, major protein-associated loci, such as the cqSeed protein-003 locus on chromosome 20, have been further fine-mapped and linked to candidate genes, providing valuable resources for molecular breeding of high-protein soybean varieties (Fliege et al., 2022). In addition, integrated analyses of soybean protein and oil QTLs have revealed genomic hotspots and candidate regions controlling seed nutritional quality (Li et al., 2019). Similar approaches have also been applied in other legumes, including pea and chickpea, where QTL mapping and genomic analyses have contributed to the identification of loci controlling seed size, nutritional composition, and processing-related traits (Verma et al., 2015; Klein et al., 2020). These studies demonstrate that seed quality traits are generally controlled by multiple genetic loci with complex environmental interactions. However, compared with soybean and other well-studied legumes, the genetic architecture underlying comprehensive seed quality traits in mungbean remains poorly characterized.

Despite the agronomic and nutritional importance of mungbean, the genetic loci controlling seed quality traits remain largely unidentified, creating a critical knowledge gap that restricts targeted breeding. Quantitative trait loci (QTLs) sequencing offers a powerful solution to unravel this genetic architecture, providing the foundation for precision breeding and developing superior mungbean varieties. Linkage analysis using biparental populations has been a fundamental approach for dissecting the genetic architecture of complex traits in crops. By constructing recombinant inbred line (RIL) or F_2_ populations, researchers can identify QTLs associated with key agronomic and quality-related characteristics. In mungbean, several studies have successfully employed this strategy to uncover QTLs for important traits. In a recent study, five stable QTLs for hundred-seed weight (HSW) across multiple environments were identified using a RIL population of 200 lines genotyped through whole-genome resequencing (Han et al., 2025). Notably, the major QTL *qHSW1* on chromosome 1 consistently explained 16.65 - 26.15% of phenotypic variation. Through fine mapping, the candidate region was narrowed to a 506-kb interval, providing valuable markers for marker-assisted breeding of high-yield varieties (Han et al., 2025). Another study successfully identified two major QTLs on chromosome 7: *qSPAD-7–1* controlling chlorophyll content and *qTric-7–2* regulating trichome density in mungbean using a RIL population (Kumari et al., 2024). Through comprehensive functional analysis, 35 candidate genes were identified, including three key regulators of trichome density and three crucial genes for chlorophyll biosynthesis. This discovery not only enhances our understanding of mungbean’s physiological traits but also provides valuable genetic markers for developing improved varieties (Kumari et al., 2024).

Despite the importance of mungbean seed quality, the genetic architecture underlying key seed quality traits, including protein, starch, oil, and moisture content, remains poorly understood. Previous studies in mungbean have mainly focused on agronomic traits such as yield, hundred-seed weight, and plant architecture, whereas systematic genetic dissection of seed quality traits is still limited. In this study, we employed integrated QTL sequencing approaches combining high-density genotyping and multi-environment phenotyping in a RIL population derived from parents contrasting in seed quality traits. By combining multiple complementary QTL mapping methods, we aimed to identify robust genomic regions associated with seed quality variation and provide valuable genetic resources for marker-assisted breeding and future functional characterization of candidate genes.

## 2 Materials and methods

### 2.1 Plant materials and phenotyping

A RIL population containing 218 lines was developed via single-seed descent from a cross between two phenotypically contrasting mungbean accessions: Zhonglv1, characterized by higher protein content, higher starch content, lower oil content, and higher moisture content, and HB211, which exhibited lower protein content, lower starch content, higher oil content, and lower moisture content. This germplasm resource is maintained in Tai yuan Normal University and Center for Agricultural Genetic Resources Research in Shanxi Agricultural University.

Phenotypic evaluations were performed during the 2022 growing season at three geographically dispersed locations in Shanxi province, China: Lvliang (LL, 37°55’ N, 112°15’ E), Jinzhong (JZ, 36°55’ N, 111°47’ E), and Yuncheng (YC, 34°07’ N, 110°58’ E). The RIL population and parental lines were cultivated in a randomized complete block design with three replications. Planting was arranged in three-row plots measuring 2 m in length, with inter-row spacing of 30 cm and intra-row spacing of 20 cm, consistent with standard agronomic protocols.

Mungbean seed quality traits—including protein, starch, oil, and moisture content—were evaluated according to the established criteria outlined in the Descriptors and Data Standards for Mungbean Germplasm Resources. After harvest, all seeds were dried under identical ambient conditions (25°C) with continuous ventilation until reaching a constant weight. The dried seeds were stored in sealed bags at room temperature until seed quality evaluation. For quantification, approximately 5 g of intact, mature, and dry seeds were homogenized using a Mini-Mill (Thomas Wiley, Swedesboro, NJ) fitted with a 20-mesh screen. The resulting powder was subsequently analyzed via near-infrared (NIR) spectroscopy employing a FOSS NIR System 6500 spectrophotometer. To ensure measurement reproducibility and precision, three independent biological replicates were processed (Baianu et al., 2012). The broad-sense heritability (*h^2^*) was calculated according to the method described by Hallauer et al. (2010), using the following formula:


$$h^{2}=\frac{\sigma_{g}^{2}}{\sigma_{g}^{2}+\sigma_{ge}^{2}/n+\sigma_{\epsilon }^{2}/rn}$$

### 2.2 Genomic DNA isolation and whole-genome sequencing

Genomic DNA was extracted from two-week-old seedling leaves of both parental lines and the 218 RIL lines, with five biological replicates, employing the Plant Genomic DNA Kit (TIANGEN) according to the manufacturer’s instructions. For each sample, a minimum of 2 µg of high-integrity genomic DNA was utilized for library construction. Sequencing libraries were prepared to generate 150-bp paired-end reads with average insert sizes of approximately 350 bp. High-throughput sequencing was performed on the DNBSEQ-T7 platform. Raw sequencing data were processed to remove low-quality reads and residual adapter contaminants using SOAPnuke v2.0.5, thereby ensuring high-quality data for subsequent genomic analyses (Chen et al., 2018).

### 2.3 SNPs identification

Sequence alignment was performed using BWA (v0.7.17) (Li and Durbin, 2009a) to map the filtered reads from the parental lines against the chromosome-level reference genome of mungbean M5311 (Li et al., 2022). The generated SAM files were subsequently converted to BAM format and sorted through SAMtools (v1.9) (Li et al., 2009b). Single nucleotide polymorphisms (SNPs) was identified utilizing GATK (v4.1.8) with stringent quality control thresholds: QD < 2.0, MQ < 40.0, FS > 60.0, SOR > 3.0, MQRankSum < -12.5, and ReadPosRankSum < -8.0 (McKenna et al., 2010). To ensure robust variant detection, three sequential filtering criteria were applied: 1) biallelic polymorphisms demonstrating clear segregation between parental genotypes, 2) minimum read depth of four high-quality reads per locus, and 3) compliance with the expected 1:1 Mendelian segregation ratio validated by *Chi*-square test (*p* < 0.001). Furthermore, co-segregating markers exhibiting complete linkage disequilibrium were systematically removed from the dataset (McKenna et al., 2010). This optimization strategy effectively reduced marker redundancy while preserving genetic map integrity, thereby enhancing computational efficiency without compromising map resolution.

### 2.4 Genetic map construction

Genotypic data derived from 218 RIL lines were employed for the construction of a genetic linkage map using JoinMap v4.0. Marker ordering was performed, and linkage group assignments were determined through the application of a logarithm of odds (LOD) threshold of 5.0, ensuring statistical confidence in marker grouping. Subsequently, genetic distances were estimated through the implementation of the Kosambi mapping function, which facilitates the conversion of recombination frequency into centimorgan (cM) units while accounting for crossover interference (Stam, 1993).

### 2.5 Identification of QTLs for seed quality traits

Genotypic data from the RIL population were associated with phenotypic measurements for protein, starch, oil, and moisture contents collected across three environments (LL, JZ and YC), enabling QTL analysis of seed quality traits. To enhance detection reliability, three complementary QTL mapping methods including composite interval mapping (CIM), inclusive composite interval mapping (ICIM), and multiple QTL mapping (MQM) were employed using WinQTL Cartographer (v2.5), QTL IciMapping (v4.1), and MapQTL 6, respectively. CIM, ICIM, and MQM were applied as complementary methods for QTL identification. CIM emphasizes background effect control, ICIM integrates interval mapping with regression analysis for stable detection of QTLs with different effects, and MQM incorporates marker cofactors to improve the detection power for complex quantitative traits (Van Ooijen, 2009; Silva et al., 2012; Meng et al., 2015).

Covariate-based QTL analysis was performed using QTL.gCIMapping.GUI (v2.0) implemented in R website (Meng et al., 2015). MQM analysis was conducted in MapQTL 6 using the same statistical threshold (Van Ooijen, 2009). Empirical LOD thresholds for each trait-environment combination were determined using 1,000 permutation tests at an experiment-wise significance level of *P* = 0.05 in WinQTL Cartographer (Silva et al., 2012). These permutation-derived thresholds were used as the statistical criteria for QTL detection (**Supplementary Table 1**). Considering the differences among QTL mapping software and to maintain consistency across CIM, ICIM, and MQM analyses, a uniform LOD threshold of 2.0 was applied as the minimum reporting threshold and a scanning interval of 1.0 cM were applied consistently across all methods. Detected QTLs were named according to standard genomic nomenclature conventions. QTLs associated with the same trait were considered the same locus when their confidence intervals overlapped across different environments or mapping methods, whereas overlapping QTLs controlling different traits were retained separately because they may represent pleiotropic effects.

## 3 Results

### 3.1 Evaluation of mungbean seed quality traits in parental lines and the RIL population

**Figure 1:**
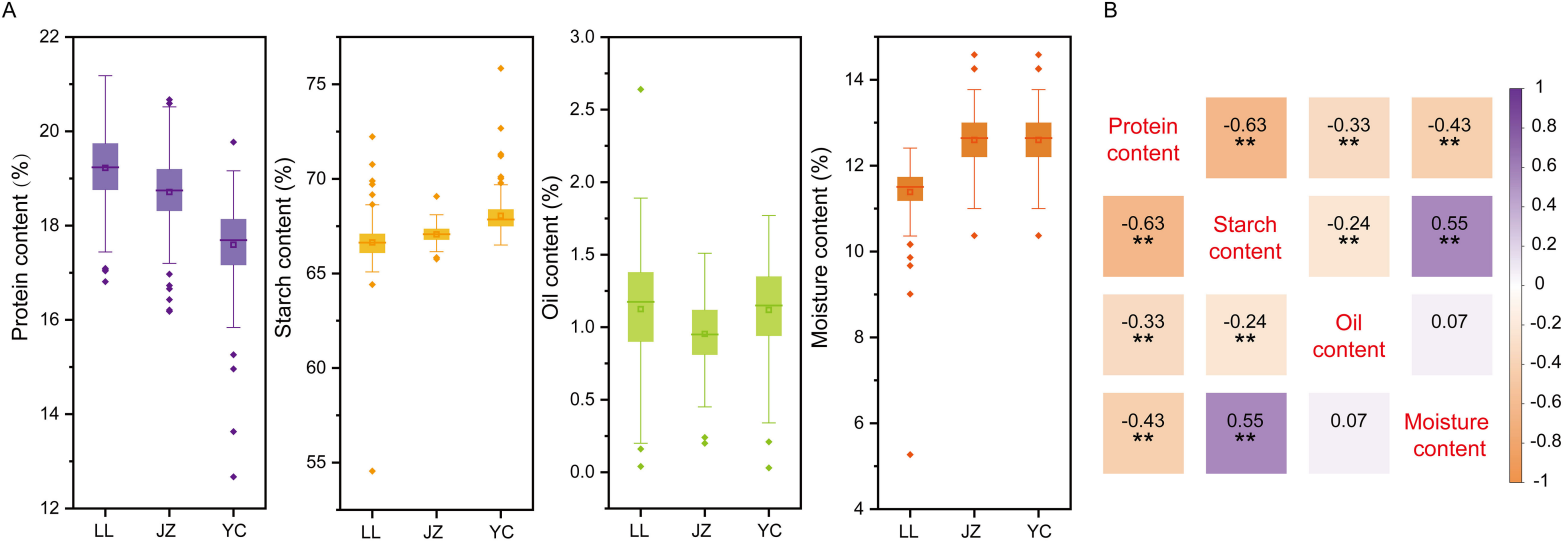
Phenotypic variation and correlation analysis of seed quality traits. **(A)** Phenotypic variability of protein, starch, oil, and moisture content in the RIL population (Zhonglv1 × HB211) evaluated across three environments: Lvliang (LL), Jinzhong (JZ), and Yuncheng (YC). **(B)** Pairwise correlations among the four traits across all environments, represented by Pearson correlation coefficients. Statistical significance was determined using a two-tailed *t*-test (*p < 0.05; **p < 0.01).

Phenotypic evaluations of seed quality traits were performed under three distinct environments using the RIL population derived from a cross between Zhonglv1 and HB211. Phenotypic differences were observed between the two parental lines: Zhonglv1 exhibited higher protein, starch, and moisture content but lower oil content relative to HB211 (**Table 1**). The broad-sense heritability values ranged from 73.58% to 83.01%, indicating genetic effects contributed substantially to the observed phenotypic variation of these traits (**Table 1**). Within the RIL population, substantial segregation was detected for all traits, with standard deviations ranging from 0.21 to 1.29 (**Table 1**; **Figure 1A**). Normality of trait distributions was evaluated using the Kolmogorov-Smirnov *test*. Most phenotypic datasets conformed to normal distribution parameters, with absolute skewness and kurtosis values below 1.0 (**Table 1**). These findings are consistent with the polygenic inheritance patterns typical of quantitative traits (Sun and Wu, 2015). Furthermore, correlation analyses among the four seed quality traits were conducted using phenotypic data across the three environments. Significant correlations among several traits were identified: starch content was positively correlated with moisture content but negatively correlated with oil content. Protein content was negatively associated with the other three traits examined in this study (**Figure 1B**).

**Table 1:** Multi-environment phenotypic performance of seed quality traits in parental lines and the RIL population.

Trait	Env.	Parent		Population						
		P1	P2	Min	Max	Mean	*SD*	Skewness	Kurtosis	*h^2^*/%
Protein Content (%)	LL	19.98 ± 0.11	20.27 ± 0.20^*^	16.81	21.18	19.22	0.81	-0.24	0.10	77.09
	JZ	18.99 ± 0.15	19.30 ± 0.14^*^	16.18	20.67	18.71	0.74	-0.45	1.25	
	YC	16.98 ± 0.09	17.24 ± 0.12^*^	12.67	19.77	17.59	0.87	-1.68	7.20	
Starch Content (%)	LL	59.60 ± 2.08	66.85 ± 1.17^***^	54.55	72.23	66.64	1.29	-3.34	39.50	73.58
	JZ	66.41 ± 0.31	67.13 ± 0.42^*^	65.76	69.07	67.06	0.45	0.26	1.35	
	YC	69.08 ± 0.12	67.39 ± 0.10^*^	66.50	75.84	68.05	1.02	3.46	20.69	
Oil Content (%)	LL	1.23 ± 0.12	0.86 ± 0.22^*^	0.04	2.64	1.12	0.39	-0.23	0.66	76.13
	JZ	0.98 ± 0.05	0.89 ± 0.05^*^	0.20	1.51	0.95	0.21	-0.28	0.48	
	YC	0.93 ± 0.06	0.79 ± 0.05^*^	0.03	1.77	1.12	0.31	-0.58	0.36	
Moisture Content (%)	LL	11.24 ± 0.25	11.74 ± 0.27^*^	5.27	12.41	11.38	0.63	-5.14	44.58	83.01
	JZ	10.06 ± 0.12	10.32 ± 0.18^*^	8.37	12.10	10.41	0.72	-0.29	-0.22	
	YC	12.58 ± 0.13	12.86 ± 0.12^*^	10.37	14.58	12.60	0.62	-0.08	0.89	

Three environments were Lvliang (LL), Jinzhong (JZ), and Yuncheng (YC) during the 2022 growing season. The values for parental lines are presented as mean ± SD. *, ** and *** indicate significant differences between the two parents at *p* < 0.05, *p* < 0.01 and *p* < 0.001, respectively (Student’s *t*-test). P1, HB211; P2, Zhonglv1; *SD*, *standard deviation*. *h^2^*, broadsense heritability.

### 3.2 SNPs identification

Whole-genome resequencing was performed on the RIL population and its parental lines, generating a total of 1,689,241,537 high-quality reads. Average sequencing depths of 24.26× and 21.83× were achieved for Zhonglv1 and HB211, respectively, while the RIL population was sequenced at an average depth of 2.53×. Through comprehensive bioinformatic processing, 802,715 high-confidence single nucleotide polymorphisms (SNPs) were identified between the two parental lines. Subsequent filtration based on quality and distribution yielded 15,667 high-quality SNPs that were retained for genetic map construction and QTL analysis. The genetic map exhibited an average marker density of 0.31 cM between adjacent SNPs. Marker intervals varied across chromosomes, ranging from 0.19 cM on chromosome 6 to 2.78 cM on chromosome 10, reflecting differential recombination frequencies throughout the genome. Further examination revealed that chromosome 10 exhibited substantially lower marker density compared with other chromosomes, likely due to low genetic divergence between the two parental lines in these regions. Such conserved regions may lack sufficient polymorphism to facilitate effective QTL detection, which may simply reflect marker sparsity rather than the true genetic architecture (**Table 2**).

**Table 2:** Chromosomal distribution of SNP markers in the mungbean genetic linkage map.

Linkage group	Number of SNP markers	Total distance (cM)	Average distance (cM)
Chr. 1	2887.00	610.57	0.21
Chr. 2	1475.00	534.60	0.36
Chr. 3	1880.00	487.47	0.26
Chr. 4	970.00	408.57	0.42
Chr. 5	1478.00	531.32	0.36
Chr. 6	1768.00	327.99	0.19
Chr. 7	1942.00	567.60	0.29
Chr. 8	1232.00	285.39	0.23
Chr. 9	1582.00	460.58	0.29
Chr. 10	93.00	258.73	2.78
Chr. 11	360.00	331.36	0.92
Total	15667.00	4804.18	0.31

### 3.3 Identification of QTLs associated with mungbean seed quality

Three complementary QTL mapping methods (CIM, ICIM, and MQM) were employed using WinQTL Cartographer (v2.5), QTL IciMapping (v4.1), and MapQTL 6, respectively (Van Ooijen, 2009; Silva et al., 2012; Meng et al., 2015). A genome-wide LOD score threshold of ≥ 2.0 was established for declaring significant QTLs across all environments. Detailed information on 162 significant QTLs, including chromosome location, confidence interval, LOD score, and phenotypic variance explained (PVE) was provided in **Supplementary Table 2**. LOD distribution and Q-Q plot demonstrated that the test statistics were well calibrated (**Supplementary Figure 1**-**S4**).

**Figure 2:**
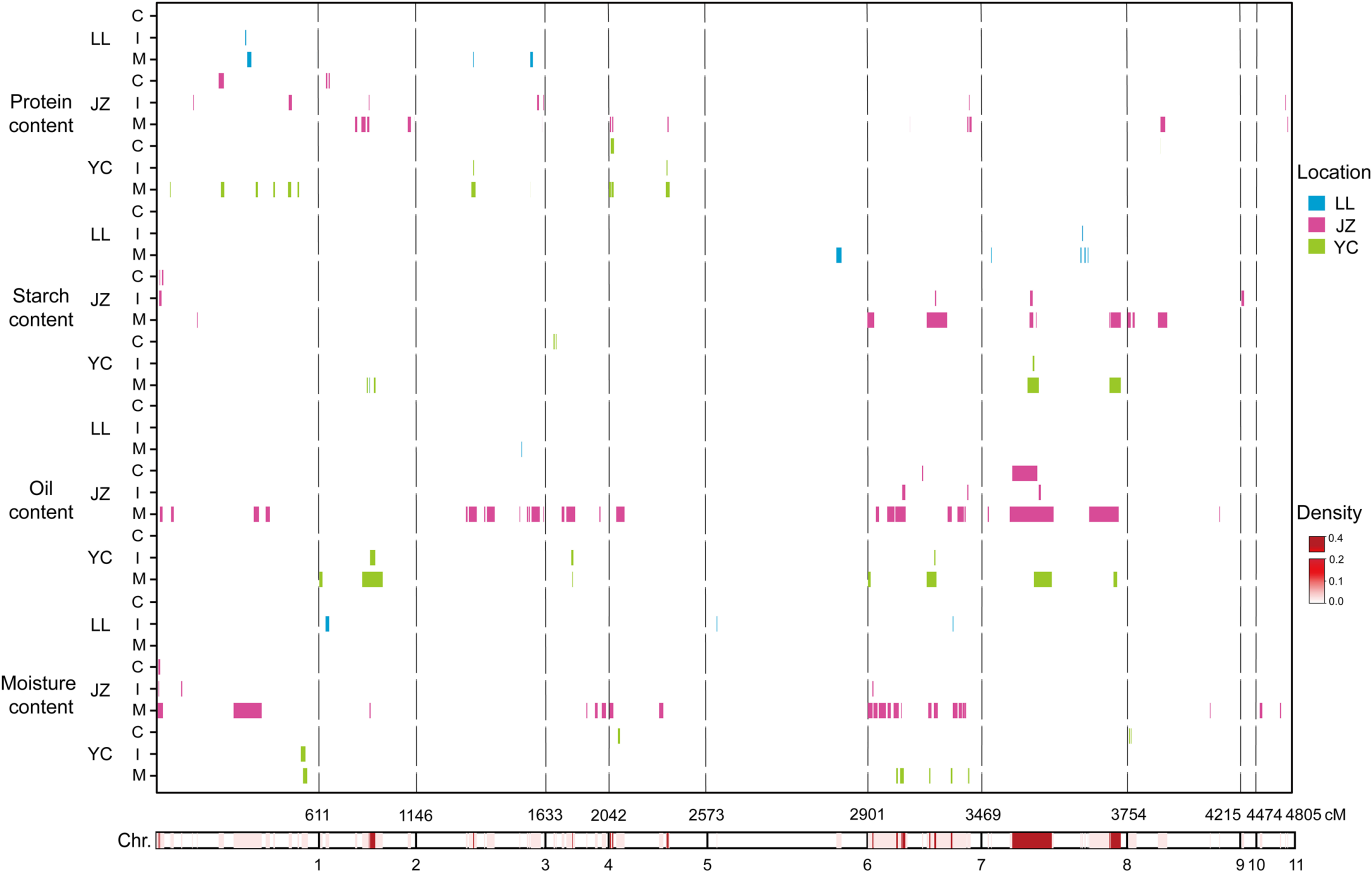
Genome-wide distribution of QTLs associated with seed quality traits in mungbean. Individual horizontal marks indicate SNP marker positions along each chromosome. The density strips at the bottom of each chromosome represent SNP marker density calculated within sliding windows across the linkage groups. Darker regions indicate higher marker density, whereas lighter regions indicate lower marker density. Mapping methods were denoted as follows: C, composite interval mapping (CIM); I, inclusive composite interval mapping (ICIM); M, multiple QTL mapping (MQM). Locations were indicated as: LL, Lvliang; JZ, Jinzhong; YC, Yuncheng.

**Figure 3:**
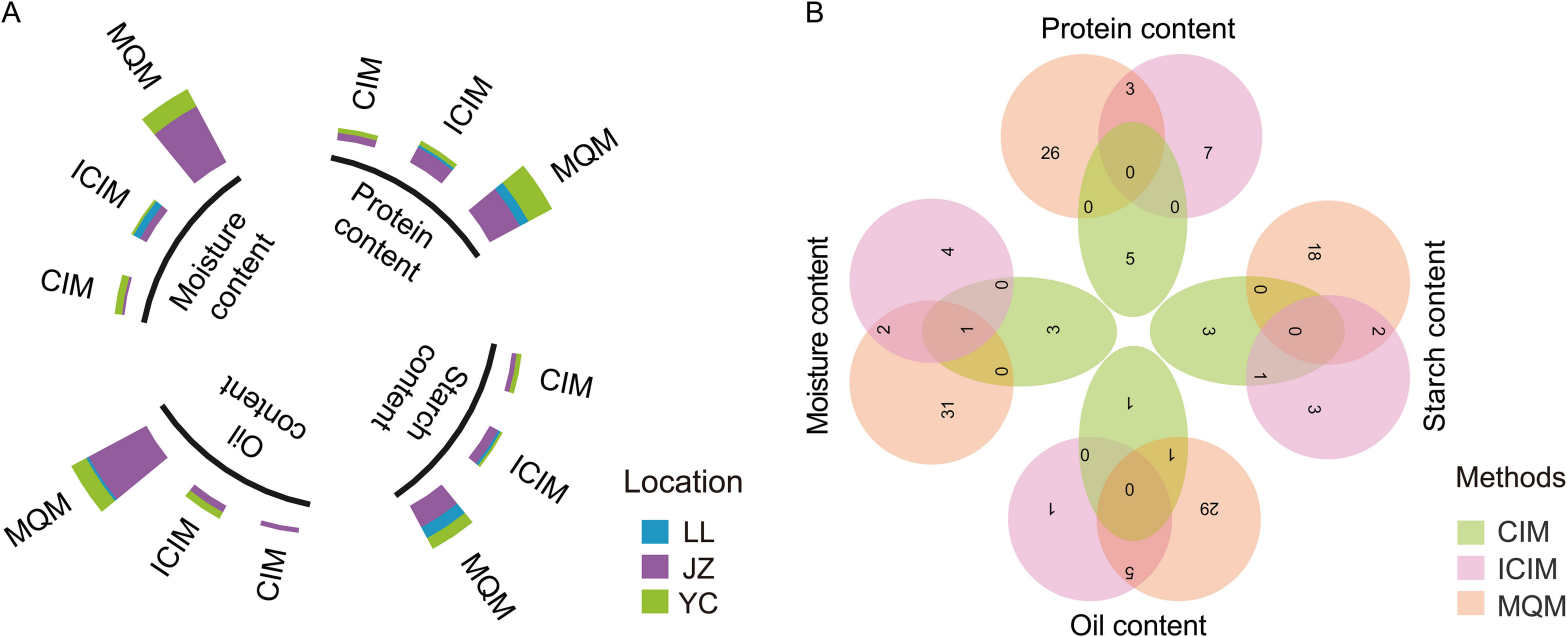
Method-specific QTLs detected across three environments. **(A)** Number of QTLs identified by composite interval mapping (CIM), inclusive composite interval mapping (ICIM), and multiple QTL mapping (MQM) in three locations: LL (Lvliang), JZ (Jinzhong), and YC (Yuncheng). **(B)** Venn diagram showing the co-localization of QTLs detected by three independent mapping methods.

Comprehensive QTL profiling revealed specific detection patterns across environments. For protein content, no loci were detected using CIM in LL, while 1 and 4 QTLs were identified via ICIM and MQM, respectively. In JZ, 3 QTLs were mapped with CIM, 7 with ICIM, and 14 with MQM. YC environment yielded 2, 2, and 11 QTLs through CIM, ICIM, and MQM. The coefficient of determination R² values for protein content-associated QTLs ranged from 3.16% to 22.14% (**Figures 2**, **3**). Starch content analysis showed method-dependent efficiencies. In LL, no CIM-based QTLs were detected, compared to 1 ICIM-derived and 5 MQM-mapped loci. JZ yielded 2, 4, and 10 QTLs via CIM, ICIM, and MQM, respectively. YC environment showed 2, 1, and 5 QTLs detected through CIM, ICIM, and MQM, respectively. Starch-related QTLs exhibited R² values from 3.89% to 11.74% (**Figures 2**, **3**). Oil content analysis revealed specific detection: LL yielded no CIM/ICIM-based QTLs but 1 MQM-mapped locus. JZ showed 2, 3, and 27 QTLs via CIM, ICIM, and MQM, respectively, while YC yielded 0, 3, and 7 QTLs. R² values spanned 4.10% to 26.40% (**Figures 2**, **3**). Moisture content profiling showed variable detection: LL yielded 3 ICIM-based QTLs but none via CIM/MQM. YC showed 3, 1, and 8 QTLs through CIM, ICIM, and MQM, respectively, while JZ yielded 1, 3, and 26 QTLs. R² values ranged from 2.45% to 16.14% (**Figures 2**, **3**). Comparative analysis confirmed MQM’s superior resolution, consistently detecting more QTLs across traits compared to CIM and ICIM (**Figures 2**, **3**).

### 3.4 Analysis of co-located QTLs and putative candidate genes

For protein content, six of the 44 detected QTLs were identified through multiple mapping methods and across environments. Among these, the locus *qPRO5–6* was consistently detected in two environments using two independent methods (**Figure 2**; **Table 3**). Within this interval, 78 genes were annotated, among which the putative candidate gene *VradChr5T0015325* was prioritized as a putative regulator of protein content based on functional annotation regarding molecular function, biological process, and cellular component, alongside comparison with previously reported genes associated with similar traits (Liu et al., 2023; Yang et al., 2023). *VradChr5T0015299.1* and *VradChr5T0015307.1* were annotated as peptidase- and proteasome activator-related genes, respectively. Similar protein metabolism-associated loci have been reported in soybean QTL studies (Bolon et al., 2010; Patil et al., 2017), indicating their potential relevance to seed protein regulation in mungbean (**Table 4**; **Supplementary Table 3**).

**Table 3:** Stable QTLs for seed quality traits identified through integrated mapping methods across multiple environments.

Trait	QTL	Chr.	Pos.	LOD	PVE	Method	Env.
Protein content	*qPRO2-5*	2	280.35 - 281.06	1.16 - 6.63	2.40-9.83	ICIM, MQM	JZ
	*qPRO3-3*	3	218.01 - 218.03	2.04 - 2.10	4.70-5.00	MQM	LL, YC
	*qPRO3-4*	3	224.17 - 225.13	2.00 - 2.50	4.43-6.00	ICIM, MQM	YC
	*qPRO5-1*	5	0.00 - 8.09	2.02 - 2.49	4.90 - 6.00	MQM	JZ, YC
	*qPRO5-2*	5	27.85 - 27.90	2.47 - 2.51	5.10 - 6.00	MQM	JZ, YC
	***qPRO5-6***	**5**	**311.54 - 316.16**	**2.55 - 3.64**	**6.20 - 8.67**	**ICIM, MQM**	**JZ, YC**
Starch content	*qSTA1-2*	1	16.70 - 17.21	2.03 - 3.01	3.89 - 11.74	CIM, ICIM	JZ
	*qSTA7-9*	7	350.86 - 351.94	2.03 - 2.98	3.89 - 6.10	ICIM, MQM	JZ
	***qSTA8-4***	**8**	**96.76 - 102.75**	**2.63 - 3.79**	**5.10 - 7.70**	**ICIM, MQM**	**JZ, YC**
	*qSTA8-11*	8	250.66 - 251.66	2.00 - 2.29	4.10 - 5.60	MQM	JZ, YC
	*qSTA8-13*	8	259.78 - 279.31	2.04 - 2.24	4.20 - 5.40	MQM	JZ, YC
Oil content	*qOIL2-6*	2	292.82 - 299.58	3.53 - 4.14	8.90 - 9.80	ICIM, MQM	YC
	*qOIL4-6*	4	189.40 - 189.43	2.01 - 2.03	4.31 - 4.90	ICIM, MQM	YC
	*qOIL7-4*	7	173.31 - 176.93	2.08 - 12.14	4.30 - 26.40	ICIM, MQM	JZ
	*qOIL7-8*	7	337.99 - 338.69	2.62 - 3.49	6.30 - 8.30	ICIM, MQM	YC
	*qOIL8-3*	8	63.36 - 110.37	3.03 - 3.60	7.30 - 11.57	CIM, MQM	JZ
	***qOIL8-5***	**8**	**105.53 - 141.07**	**2.02 - 5.40**	**4.90 - 10.50**	**ICIM, MQM**	**JZ, YC**
	*qOIL8-14*	8	265.78 - 278.31	2.01 - 5.20	4.90 - 10.40	MQM	JZ, YC
Moisture content	*qMOI1-1*	1	12.54 - 13.15	2.88 - 5.09	6.60 - 9.45	CIM, ICIM, MQM	JZ
	*qMOI7-1*	7	52.38 - 53.12	3.93 - 4.53	6.66 - 9.10	ICIM, MQM	JZ
	*qMOI7-2*	7	147.15 - 151.31	2.00 - 3.35	4.50 - 6.80	MQM	JZ, YC
	*qMOI7-3*	7	167.51 - 167.53	2.00 - 2.16	4.50 - 4.90	MQM	JZ, YC
	*qMOI7-7*	7	309.62 - 309.64	2.06 - 2.19	4.30 - 5.30	MQM	JZ, YC
	*qMOI7-10*	7	422.78 - 423.78	2.11 - 3.96	5.10 - 8.00	MQM	JZ, YC

The QTLs consistently identified across multiple environments and analytical methods are indicated in bold. Chr, chromosome; Pos, genetic position; Env, environment; LL, Lvliang; JZ, Jinzhong; YC, Yuncheng. Bold values indicate QTLs detected in at least two environments and identified by at least two QTL mapping methods.

**Table 4:** The putative candidate genes for seed quality traits annotated within co-localized QTL regions.

QTL	Chr.	Gene ID	Start	End	Annotation
*qPRO5-6*	5 5 5	*VradChr5T0015325* *VradChr5T0015307* *VradChr5T0015299*	1253101 1105982 1034566	1253839 1109471 1036054	Auxin-activated signaling pathway amino acid transport proteasome-activating activity peptidase activity
*qSTA8-4*	8	*VradChr8T0023264*	18689048	18703005	starch biosynthetic process
	8	*VradChr8T0022861*	9956267	9963099	sucrose synthase activity
*qOIL8-5*	8	*VradChr8T0022972*	12550043	12551520	Fatty acid metabolic process
	8	*VradChr8T0023123*	15964081	15972169	Long-chain fatty acid biosynthetic process
	8	*VradChr8T0023124*	15979547	15988313	Long-chain fatty acid biosynthetic process
	8	*VradChr8T0023138*	16375693	16383207	Fatty acid biosynthetic process
	8	*VradChr8T0023139*	16388729	16404098	Fatty acid biosynthetic process
	8	*VradChr8T0023190*	17429477	17431675	Acyltransferase activity
	8	*VradChr8T0023304*	19438018	19442770	Glycerol metabolic process
	8	*VradChr8T0023309*	19487095	19492786	Glycerol metabolic process
	8	*VradChr8T0023310*	19494577	19496155	Glycerol metabolic process
	8	*VradChr8T0023311*	19496744	19499099	Glycerol metabolic process
	8	*VradChr8T0023312*	19499248	19503480	Glycerol metabolic process
	8	*VradChr8T0023381*	20524170	20526604	Fatty-acyl-CoA biosynthetic process

PRO, protein; STA, starch; Chr, chromosome.

In the case of starch content, five of the 30 identified QTLs were co-localized across methods and environments. The QTL *qSTA8–4* was stably identified in two environments using two analytical approaches (**Figure 2**; **Table 3**). A total of 74 genes were annotated within this region, with two putative candidates—*VradChr8T0023264* and *VradChr8T0022861*—selected based on gene ontology terms related to critical biological processes and previously documented roles in seed starch content (**Table 4**, **Supplementary Table 3**) (Li et al., 2013; Stein and Granot, 2019).

Regarding oil content, seven of the 43 identified QTLs were co-detected through multiple methods in various environments. The locus *qOIL8–5* was consistently observed across two environments and two mapping strategies (**Figure 2**; **Table 3**). This genomic region contained 668 genes, of which 12 putative candidates—including *VradChr8T0022972*, *VradChr8T0023123*, and *VradChr8T0023124*—were identified as potential candidates associated with oil content via functional annotation and sequence alignment (**Table 4**; **Supplementary Table 3**) (Johnston et al., 1997; Sagun et al., 2023).

For seed moisture content, six of the 45 detected QTLs were co-localized through the application of multiple mapping methods and evaluation in diverse environments. However, no QTL was consistently detected across more than two environments and confirmed by two or more analytical methods (**Figure 2**; **Table 3**). This observation suggests that the genetic control of seed moisture content is highly sensitive to environmental conditions and/or involves a complex architecture dominated by numerous small-effect loci, which are inherently challenging to detect consistently with limited population size and statistical power (Bruno et al., 2017; Wang et al., 2022).

In addition to trait-specific QTLs, several overlapping QTL regions were identified between starch and oil contents. For example, *qSTA7–2* overlapped with *qOIL7-8*, *qSTA8–4* overlapped with *qOIL8-3*, and *qSTA8–13* overlapped with *qOIL8-14* (**Table 3**). These overlapping regions suggest potential pleiotropic effects, indicating that some genomic loci may simultaneously influence starch and oil accumulation in mungbean seeds.

## 4 Discussion

As a climate-resilient and protein-rich legume, mungbean is regarded as an essential crop for enhancing nutritional security and promoting sustainable agricultural systems. The elucidation of QTLs associated with seed quality traits represents a critical step toward understanding the genetic mechanisms controlling these traits and enables marker-assisted breeding through the discovery of pivotal genomic intervals. Although substantial progress has been made in characterizing the genetic base of seed quality in staple crops such as rice, soybean, and maize (Jadhav et al., 2024; Selvan et al., 2025; Zhang Y. et al., 2025), the genomic regions underlying seed quality in mungbean have received limited attention. Linkage-based analyses have been widely employed to dissect complex quantitative traits in numerous crops (Kassem, 2025; Tong et al., 2025; Zhang X. et al., 2025).

In this study, the polygenic nature of these traits was substantiated by normally distributed phenotypic values for protein, starch, oil, and moisture content (**Table 1**). By integrating three complementary QTL mapping methods (CIM, ICIM, and MQM) within a RIL population (N = 218), a total of 162 loci were identified: 44 for protein content, 30 for starch content, 43 for oil content, and 45 for moisture content (**Figure 2**), underscoring the multigenic genetic architecture governing mungbean seed quality.

By comparing the QTL intervals identified in this study with previously reported mungbean QTLs, several overlapping genomic regions were identified. For example, *qPRO2–5* overlapped with reported yield per plant (YP)-associated loci *qYP2-2*. Moreover, *qOIL8–5* overlapped with pod length-associated loci *qPL8-4*, and pod length is an important agronomic trait closely associated with yield performance (Yang et al., 2025). These co-localizations indicate that certain genomic regions may contribute simultaneously to seed quality and yield-related traits, possibly reflecting pleiotropic effects or linked genetic factors. Notably, several co-localized QTLs for each trait were consistently detected across environments and analytical methods. The integration of multi-environment phenotyping with multiple QTL mapping strategies provides a more reliable framework for dissecting the genetic basis of complex seed quality traits. Unlike individual candidate genes, stable QTLs provide robust genetic resources that can be directly utilized for marker-assisted selection. Several co-localized QTL regions detected for different seed quality traits suggest potential genetic relationships among nutritional components. These findings provide crucial genetic insights and lay the groundwork for molecular breeding strategies aimed at improving seed quality in mungbean.

### 4.1 Identification of putative candidate genes associated with protein content

The availability of high-resolution genome assemblies and pangenome resources enhances the identification of putative candidate genes within QTL regions (Li et al., 2025). Based on integrated gene annotation and extensive review of existing study, one putative candidate gene, *VradChr5T0015325*, was identified within the co-localized QTL *qPRO5-6* (**Table 4**). *VradChr5T0015325* is implicated in the auxin-activated signaling pathway and is hypothesized to play a role in amino acid transport. The established study indicated that auxin signaling participates in the regulation of seed storage protein (SSP) accumulation (Gazzarrini et al., 2004; Yang et al., 2023). In addition, members of the LAFL network (including LEC1, ABI3, FUS3, and LEC2) have been reported to play important role in controlling the expression of SSP genes during seed maturation (Yang et al., 2023). Therefore, it is postulated that *VradChr5T0015325* may represent a putative candidate involved in seed protein regulation. Whether it directly regulates storage protein accumulation in mungbean requires further functional validation. VradChr5T0015299.1 exhibits peptidase activity. In soybean, multiple QTLs associated with the regulation of seed storage protein degradation have been identified through the screening of peptidase-related genes (Bolon et al., 2010). VradChr5T0015307.1 possesses proteasome activator activity. In soybean, several QTLs involved in proteasome-mediated seed protein processing have been revealed (Patil et al., 2017). These findings suggest that these genes may play potential roles in protein regulation in mungbean.

### 4.2 Analysis of putative candidate genes associated with starch content

The co-located QTL, *qSTA8-4*, underwent gene annotation and exhaustive studies, leading to the identification of two putative candidate genes (**Table 4**). Notably, *VradChr8T0023264* was specifically selected due to its direct involvement in the starch biosynthetic process. It is widely recognized that genes encoding enzymes or regulators within this pathway—such as starch synthases, branching enzymes, or ADP-glucose pyrophosphorylases—have been well-documented to modulate starch content in storage organs. Therefore, *VradChr8T0023264* is postulated to influence starch accumulation in mungbean seeds through its role in catalyzing or regulating key steps of starch biosynthesis.

Moreover, *VradChr8T0022861* was annotated as sucrose synthase (SuSy), an enzyme previously implicated in carbon allocation and starch biosynthesis in storage tissues (Li et al., 2013; Stein and Granot, 2019). This prioritization is strongly supported by extensive literature demonstrating the fundamental role of SuSy in starch biosynthesis, particularly in sink tissues such as seeds (Stein and Granot, 2019). Given its annotation and pivotal position in the sucrose-to-starch metabolic pathway, *VradChr8T0022861* is hypothesized to modulate starch content in mungbean seeds by regulating substrate availability for starch biosynthetic enzymes.

### 4.3 The putative candidate gene analysis related to oil content

Through the process of gene annotation and an extensive review of the relevant literature, 12 putative candidate genes were pinpointed within the co-located QTL (*qOIL8-5*). Seed oil accumulation is a complex metabolic process highly dependent on the efficient conversion of photosynthetic carbon into triacylglycerols (TGs) (Sagun et al., 2023). In seeds, sucrose serves as the primary carbon source imported into developing embryos, where it is channeled through glycolysis and the oxidative pentose phosphate pathway to generate precursors for *de novo* fatty acid synthesis (FAS) (Johnston et al., 1997). This process occurs primarily in plastids, where acetyl-CoA carboxylase (ACCase) catalyzes the first committed step, producing malonyl-CoA for iterative elongation by fatty acid synthase complexes (Sagun et al., 2023). Subsequent elongation, desaturation, and export of fatty acids to the endoplasmic reticulum enable TG assembly through the coordinated action of acyltransferases and glycerol-3-phosphate pathways (Sagun et al., 2023). Among the 12 putative candidate genes, *VradChr8T0023123* and *VradChr8T0023124* were annotated with functions related to long-chain fatty acid biosynthesis. *VradChr8T0023138* and *VradChr8T0023139*, associated with general fatty acid biosynthetic process, suggesting their potential involvement in fatty acid metabolism (Sagun et al., 2023). The putative candidate *VradChr8T0023190*, linked to acyltransferase activity, may encode enzymes such as diacylglycerol acyltransferase (DGAT). Overexpression of *DGAT* overexpression is an effective strategy for enhancing oil accumulation (Sagun et al., 2023). Moreover, five genes (*VradChr8T0023304*, *VradChr8T0023309*, *VradChr8T0023310*, *VradChr8T0023311*, and *VradChr8T0023312*) are implicated in glycerol metabolic process, reflecting their potential roles in supplying glycerol backbones for TG formation (Sagun et al., 2023). Finally, *VradChr8T0023381*, involved in fatty-acyl-CoA biosynthetic process, may participate in the activation and cytosolic elongation of fatty acids prior to TG synthesis (Sagun et al., 2023). However, the functional characterization of these putative candidates and their orthologs in closely related species remains limited, further experimental validation is required to clarify their precise roles in regulating seed oil deposition in mungbean.

### 4.4 Identification of QTLs using multiple mapping approaches

The integration of high-density genotyping enables the identification of stable QTLs across environments (Zhang et al., 2024b). QTLs associated with seed quality traits in mungbean were identified through the integrated application of three complementary methods: CIM, ICIM, and MQM. Each method exhibits distinct advantages and limitations in dissecting complex genetic architectures, which was revealed in our previous study on the identification of QTLs for mungbean yield traits (Yang et al., 2025). CIM is widely recognized for its ability to control background genetic effects by incorporating cofactor markers, thereby enhancing the accuracy and resolution of QTL localization (Silva et al., 2012). In contrast, ICIM employs a combination of interval mapping and stepwise regression, offering a balanced strategy for detecting both major and minor effect QTLs. However, this method requires careful parameter optimization to minimize false positives (Meng et al., 2015). MQM, which simultaneously incorporates multiple QTLs into a unified genetic model, demonstrates superior performance in analyzing complex trait architectures. Nevertheless, it is often computationally demanding and sensitive to initial model specifications (Van Ooijen, 2009).

In the present study, MQM detected the highest number of QTLs, a proportion of which were consistently identified across both CIM and ICIM (**Figure 3**), underscoring the reliability of these genomic regions. MQM incorporates marker cofactors as background controls, thereby reducing the influence of linked genetic effects and residual variation and improving the statistical power for QTL detection (Van Ooijen, 2009). Consequently, MQM is generally more sensitive to loci with relatively small effects compared with CIM and ICIM. In this study, the higher number of QTLs detected by MQM may be attributed to its increased ability to capture minor-effect loci that contribute to phenotypic variation. However, most MQM-specific QTLs exhibited relatively low PVE values (**Supplementary Table 2**), suggesting that these loci mainly represent small-effect genetic factors underlying complex seed quality traits. Nevertheless, each method also revealed unique QTLs that were not detected by the other approaches. This observation implies that the genetic architecture governing seed quality traits in mungbean is complex, with certain QTLs being method-specific due to variations in statistical power, model assumptions, or sensitivity to genetic background. The combined application of CIM, ICIM, and MQM provided a robust and comprehensive framework for QTL identification. By integrating the strengths of each method, the limitations inherent to individual approaches were effectively mitigated. The overlapping QTLs confirmed by multiple methods enhance confidence in these genomic regions, while uniquely detected QTLs emphasize the necessity of employing diverse analytical strategies to fully capture underlying genetic variation. This integrated multi-method strategy not only improves the reliability of QTL detection but also facilitates a more holistic understanding of the genetic basis of seed quality traits in mungbean.

Although this study identified numerous QTLs associated with mungbean seed quality traits, the potential epistatic interactions among these loci were not investigated. Since quantitative traits are often regulated by complex genetic networks involving both additive and interactive effects, future studies incorporating QTL interaction analysis and functional validation will be necessary to further elucidate the genetic mechanisms underlying seed quality formation.

## 5 Conclusion

This study presents a comprehensive genetic analysis of seed quality traits in mungbean through integrated QTL mapping. A RIL population derived from parents contrasting in protein, starch, oil, and moisture content was evaluated across multiple environments, leading to the identification of 162 QTLs associated with these traits. Notably, three environmentally stable QTLs were consistently detected using two mapping methods, underscoring their robustness and potential utility in molecular breeding. Furthermore, putative candidate genes underlying these QTLs were identified through functional annotation and homology analysis, providing insights into possible biological mechanisms regulating seed quality. These findings substantially expand the current genetic knowledge of mungbean seed quality traits and establish a foundational resource for molecular breeding. Future work should focus on fine mapping of the stable QTLs identified, as well as functional validation of putative candidate genes. This study will facilitate the breeding of improved mungbean varieties with enhanced seed quality.

## Data Availability

The original contributions presented in the study are included in the article/**Supplementary Material**. Further inquiries can be directed to the corresponding authors.

## Supplementary material

Supplementary Table 1 Empirical LOD thresholds determined by permutation tests for QTL mapping of seed quality traits across different environments.

Supplementary Table 2 Summary of QTLs detected for seed quality traits through three mapping methods across three environments.

Supplementary Table 3 A comprehensive list of genes located within the identified QTL intervals.

Supplementary Figure 1 LOD distribution of all QTLs detected by ICIM.

Supplementary Figure 2 LOD distribution of all QTLs detected by CIM.

Supplementary Figure 3 LOD distribution of all QTLs detected by MQM.

Supplementary Figure 4 Quantile-quantile (Q-Q) plots from ICIM QTL mapping for seed quality traits.
